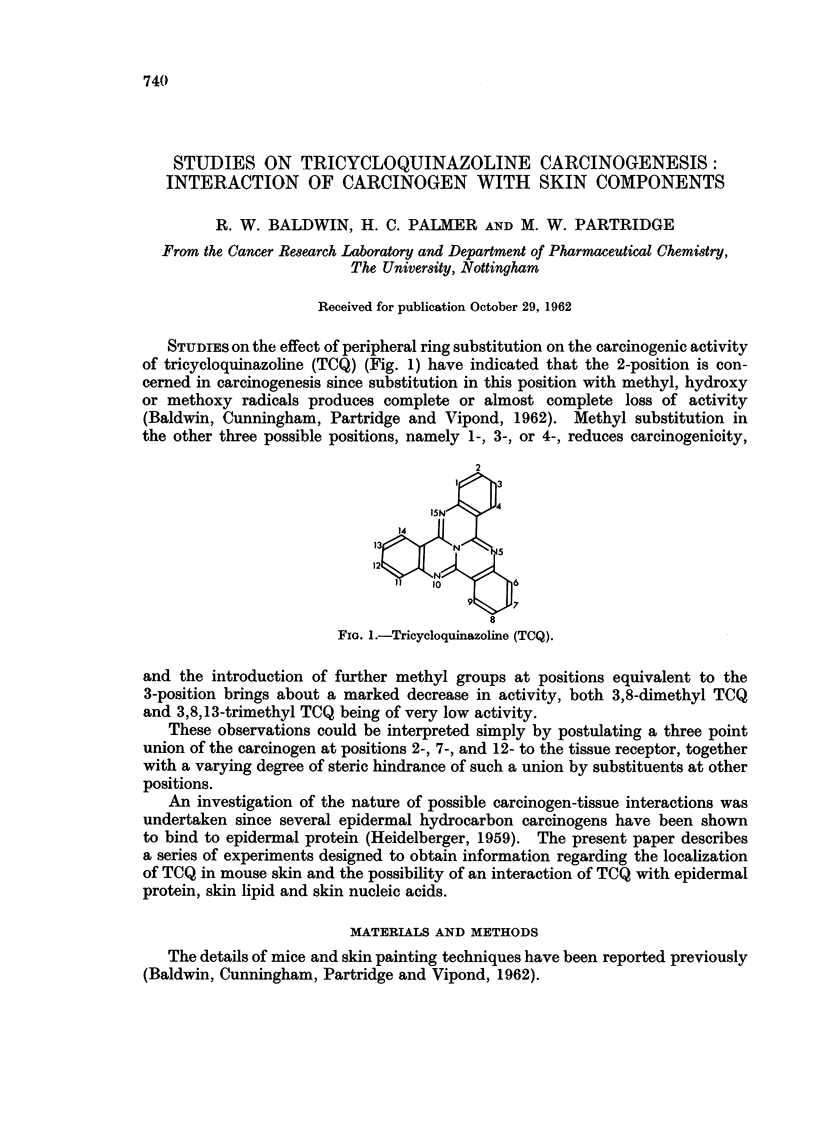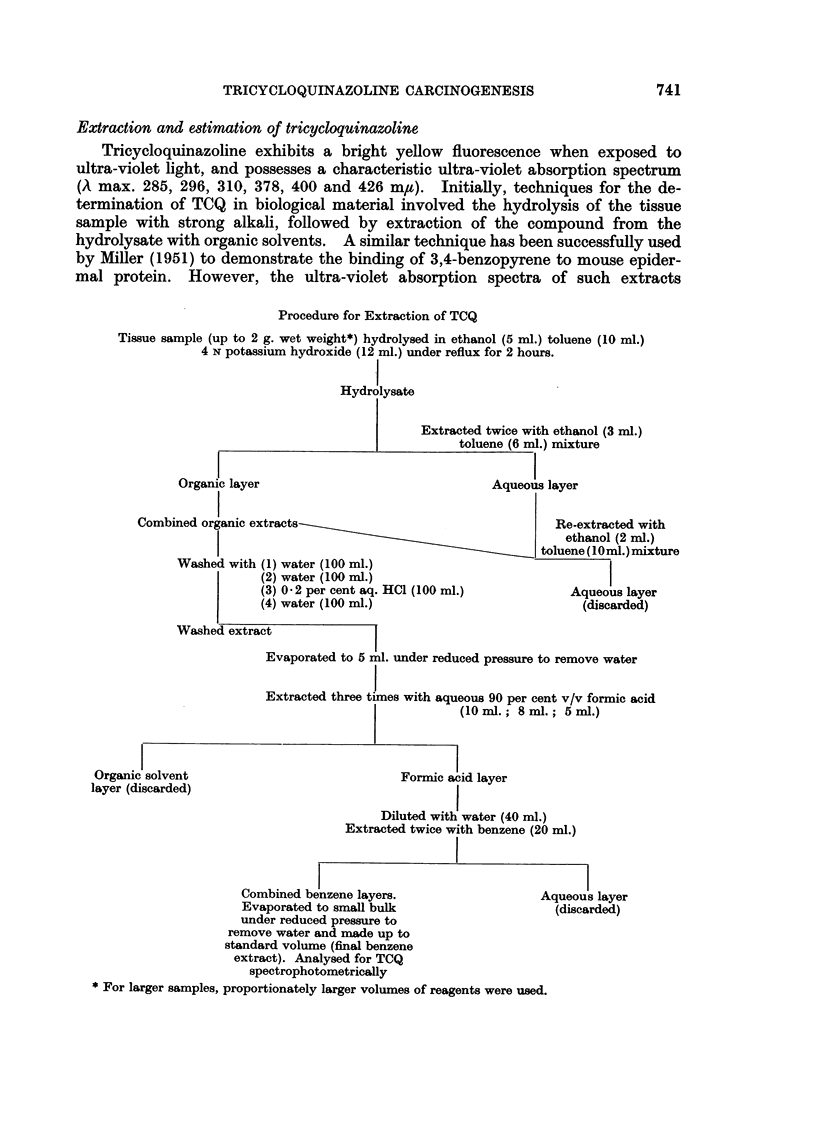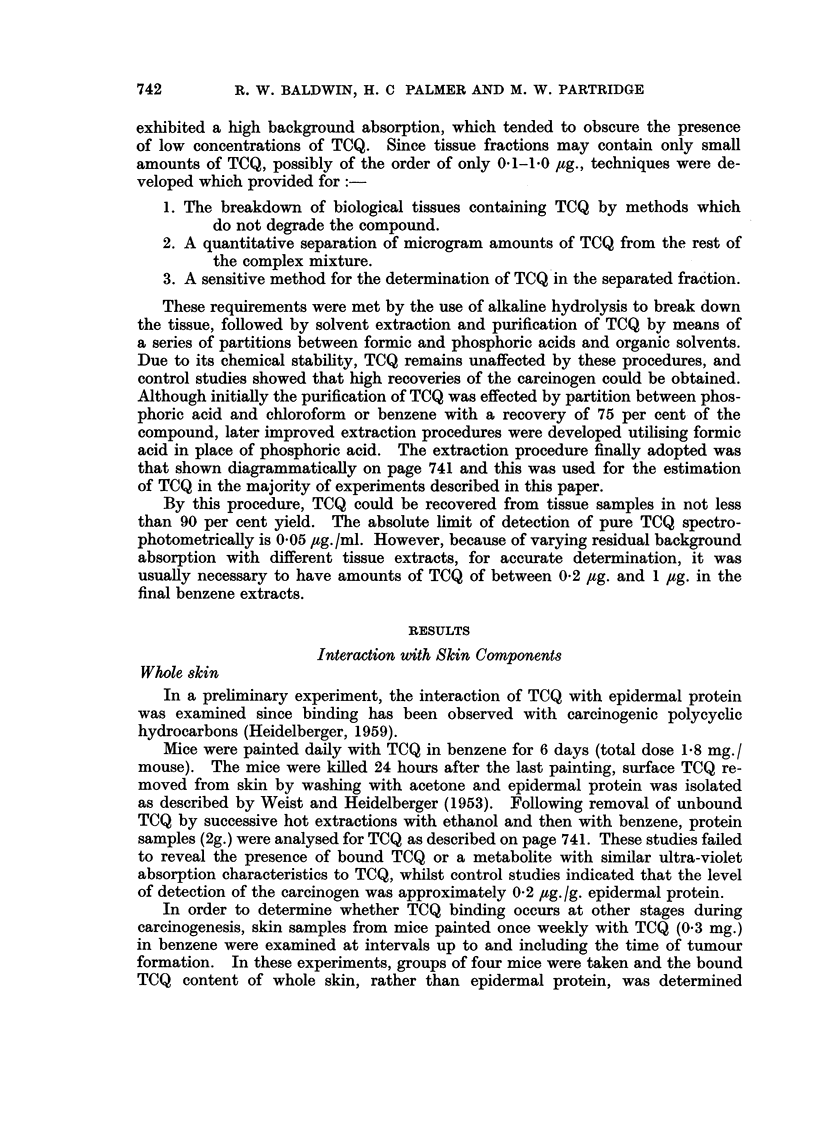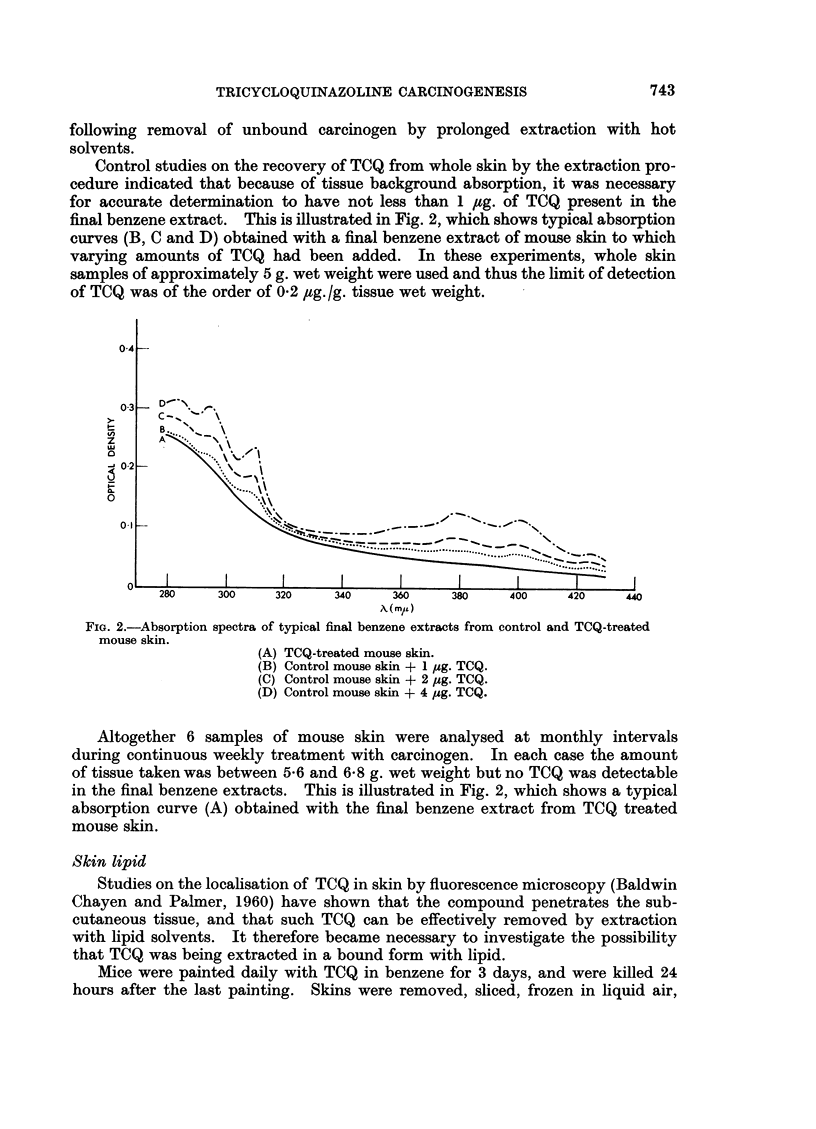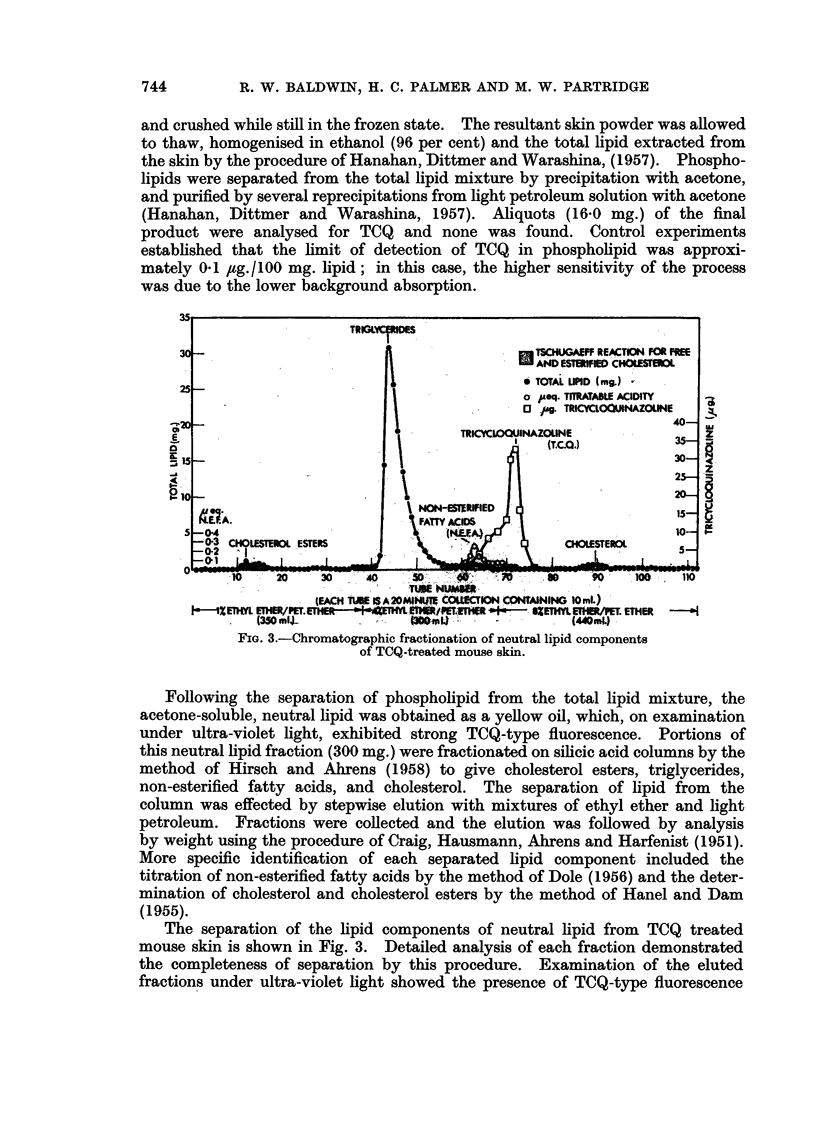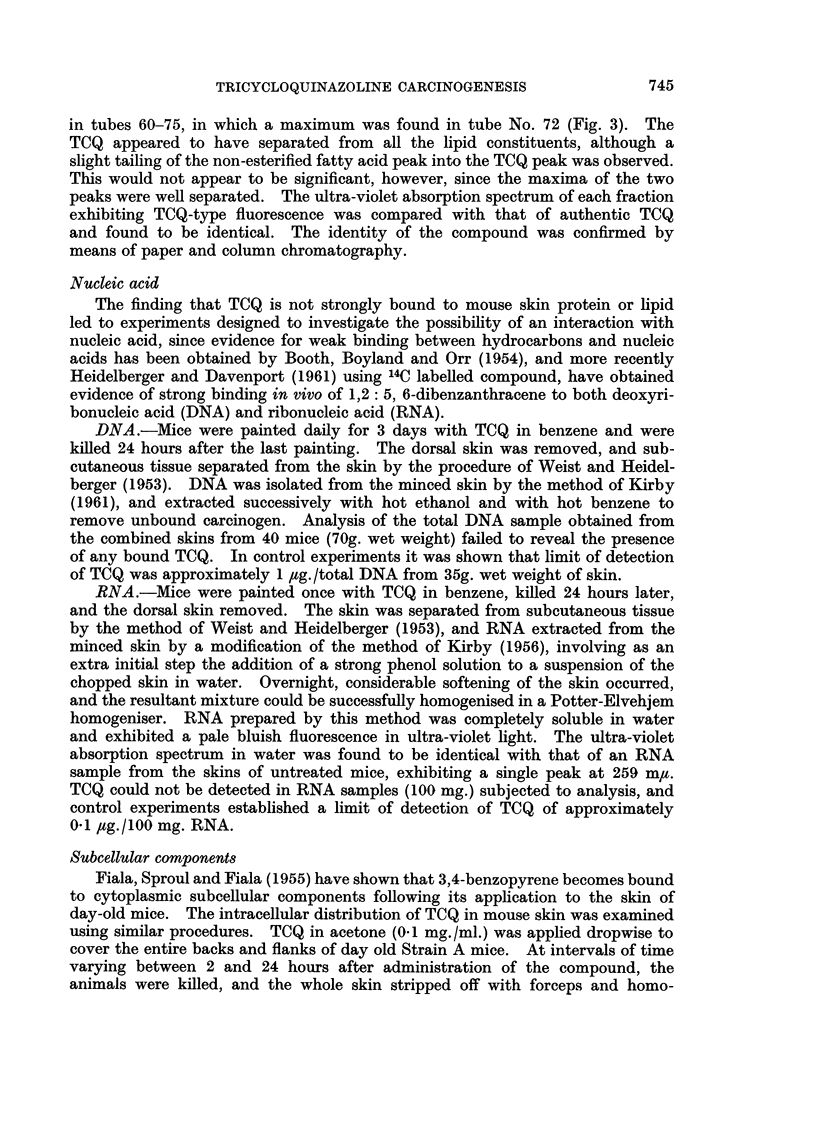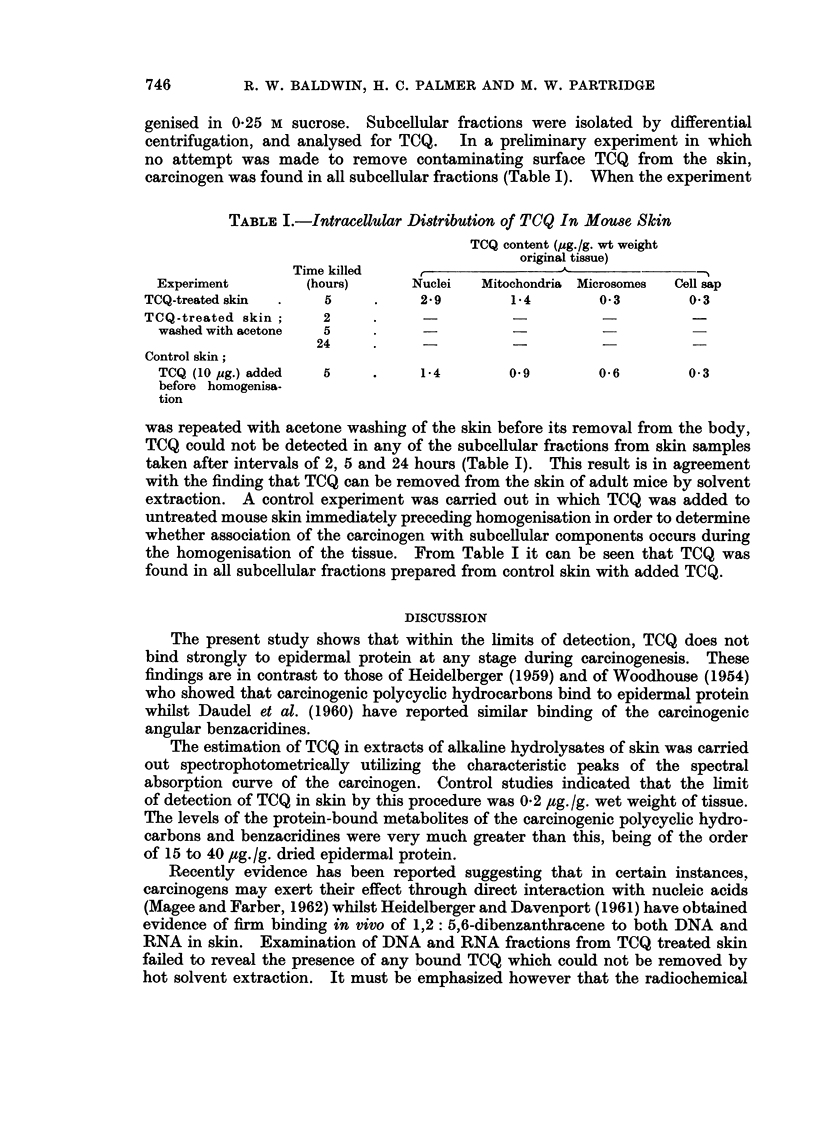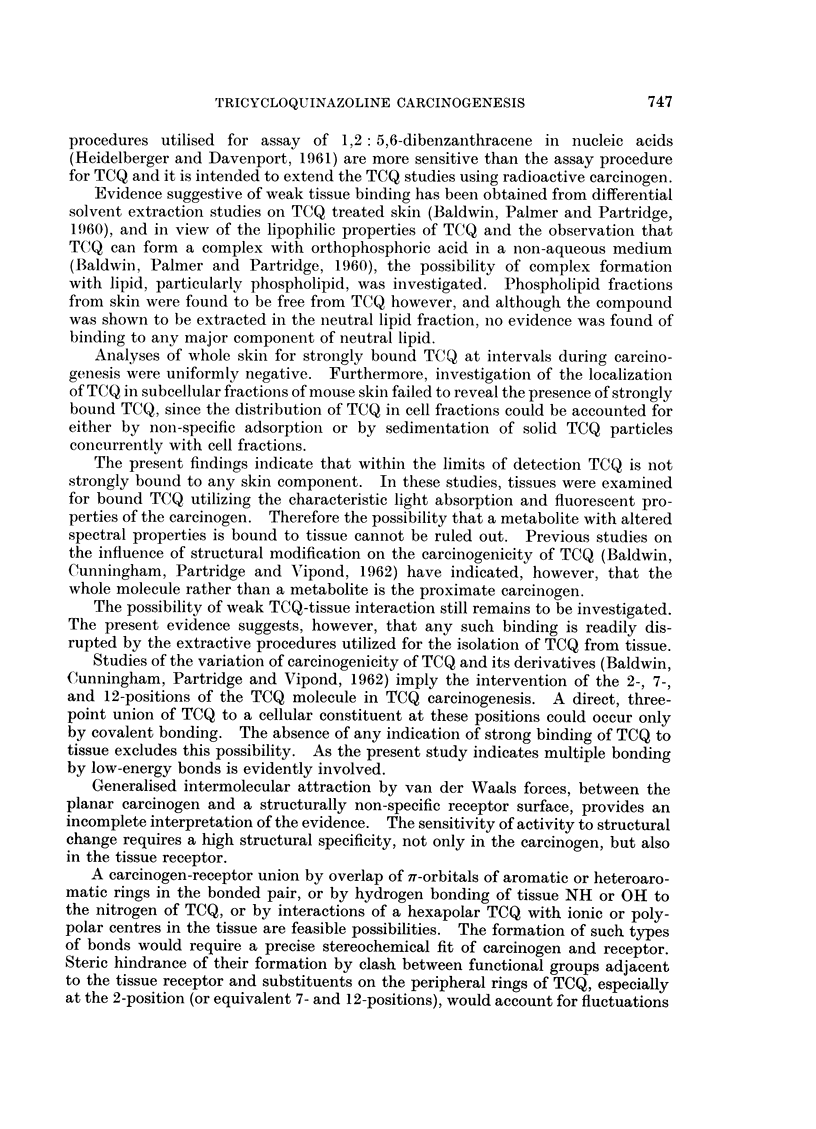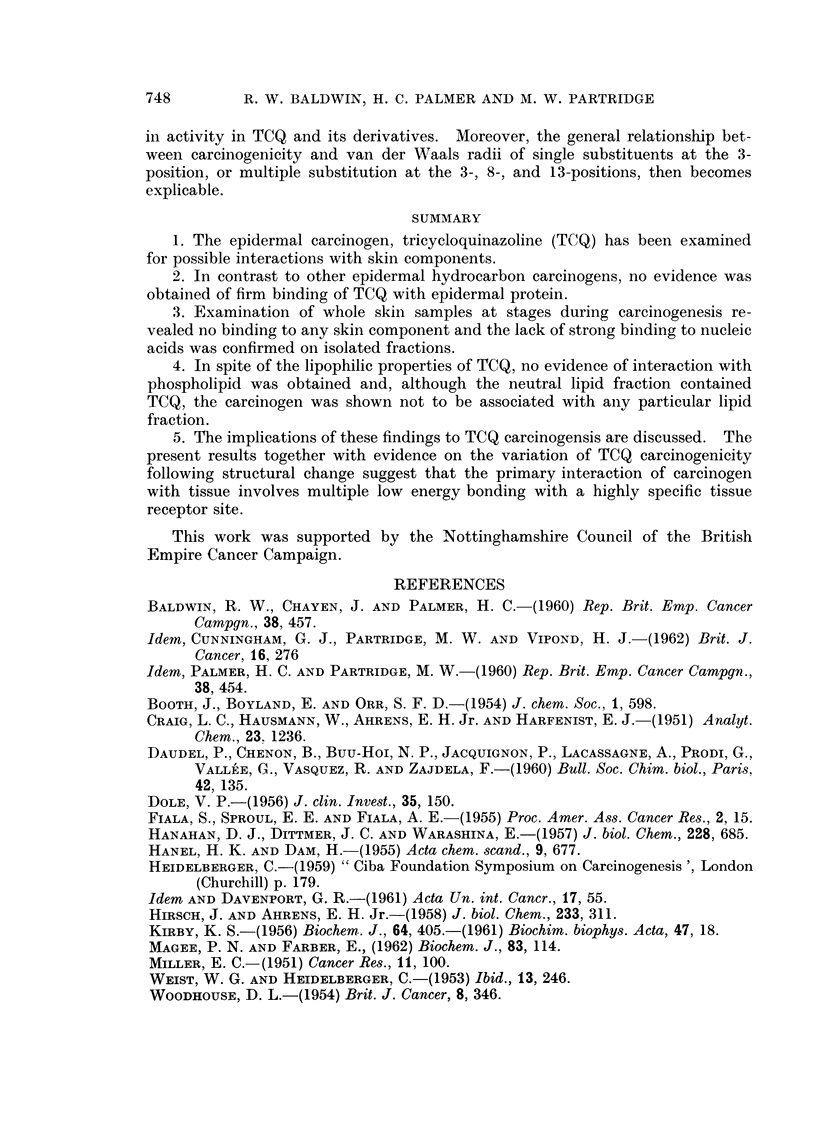# Studies on Tricycloquinazoline Carcinogenesis: Interaction of Carcinogen with Skin Components

**DOI:** 10.1038/bjc.1962.86

**Published:** 1962-12

**Authors:** R. W. Baldwin, H. C. Palmer, M. W. Partridge


### STUDIES ON TRICYCLOQUINAZOLINE CARCINOGENESIS: INTERACTION OF CARCINOGEN WITH SKIN COMPONENTS

R. W. BALDWIN, H. C. PALMER AND M. W. PARTRIDGE

*From the Cancer Research Laboratory and Department of Pharmaceutical Chemistry, The University, Nottingham*

Received for publication October 29, 1962

STUDIES on the effect of peripheral ring substitution on the carcinogenic activity of tricycloquinazoline (TCQ) (Fig. 1) have indicated that the 2-position is concerned in carcinogenesis since substitution in this position with methyl, hydroxy or methoxy radicals produces complete or almost complete loss of activity (Baldwin, Cunningham, Partridge and Vipond, 1962). Methyl substitution in the other three possible positions, namely 1-, 3-, or 4-, reduces carcinogenicity,

FIG. 1.—Tricycloquinazoline (TCQ).

and the introduction of further methyl groups at positions equivalent to the 3-position brings about a marked decrease in activity, both 3,8-dimethyl TCQ and 3,8,13-trimethyl TCQ being of very low activity.

These observations could be interpreted simply by postulating a three point union of the carcinogen at positions 2-, 7-, and 12- to the tissue receptor, together with a varying degree of steric hindrance of such a union by substituents at other positions.

An investigation of the nature of possible carcinogen-tissue interactions was undertaken since several epidermal hydrocarbon carcinogens have been shown to bind to epidermal protein (Heidelberger, 1959). The present paper describes a series of experiments designed to obtain information regarding the localization of TCQ in mouse skin and the possibility of an interaction of TCQ with epidermal protein, skin lipid and skin nucleic acids.

### MATERIALS AND METHODS

The details of mice and skin painting techniques have been reported previously (Baldwin, Cunningham, Partridge and Vipond, 1962).

*Extraction and estimation of tricycloquinazoline*

Tricycloquinazoline exhibits a bright yellow fluorescence when exposed to ultra-violet light, and possesses a characteristic ultra-violet absorption spectrum ($\lambda$ max. 285, 296, 310, 378, 400 and 426 m$\mu$). Initially, techniques for the determination of TCQ in biological material involved the hydrolysis of the tissue sample with strong alkali, followed by extraction of the compound from the hydrolysate with organic solvents. A similar technique has been successfully used by Miller (1951) to demonstrate the binding of 3,4-benzopyrene to mouse epidermal protein. However, the ultra-violet absorption spectra of such extracts

Procedure for Extraction of TCQ

* For larger samples, proportionately larger volumes of reagents were used.

exhibited a high background absorption, which tended to obscure the presence of low concentrations of TCQ. Since tissue fractions may contain only small amounts of TCQ, possibly of the order of only 0·1–1·0 μg., techniques were developed which provided for :—

1. The breakdown of biological tissues containing TCQ by methods which do not degrade the compound.
2. A quantitative separation of microgram amounts of TCQ from the rest of the complex mixture.
3. A sensitive method for the determination of TCQ in the separated fraction.

These requirements were met by the use of alkaline hydrolysis to break down the tissue, followed by solvent extraction and purification of TCQ by means of a series of partitions between formic and phosphoric acids and organic solvents. Due to its chemical stability, TCQ remains unaffected by these procedures, and control studies showed that high recoveries of the carcinogen could be obtained. Although initially the purification of TCQ was effected by partition between phosphoric acid and chloroform or benzene with a recovery of 75 per cent of the compound, later improved extraction procedures were developed utilising formic acid in place of phosphoric acid. The extraction procedure finally adopted was that shown diagrammatically on page 741 and this was used for the estimation of TCQ in the majority of experiments described in this paper.

By this procedure, TCQ could be recovered from tissue samples in not less than 90 per cent yield. The absolute limit of detection of pure TCQ spectrophotometrically is 0·05 μg./ml. However, because of varying residual background absorption with different tissue extracts, for accurate determination, it was usually necessary to have amounts of TCQ of between 0·2 μg. and 1 μg. in the final benzene extracts.

### RESULTS

### *Interaction with Skin Components*

#### *Whole skin*

In a preliminary experiment, the interaction of TCQ with epidermal protein was examined since binding has been observed with carcinogenic polycyclic hydrocarbons (Heidelberger, 1959).

Mice were painted daily with TCQ in benzene for 6 days (total dose 1·8 mg./mouse). The mice were killed 24 hours after the last painting, surface TCQ removed from skin by washing with acetone and epidermal protein was isolated as described by Weist and Heidelberger (1953). Following removal of unbound TCQ by successive hot extractions with ethanol and then with benzene, protein samples (2g.) were analysed for TCQ as described on page 741. These studies failed to reveal the presence of bound TCQ or a metabolite with similar ultra-violet absorption characteristics to TCQ, whilst control studies indicated that the level of detection of the carcinogen was approximately 0·2 μg./g. epidermal protein.

In order to determine whether TCQ binding occurs at other stages during carcinogenesis, skin samples from mice painted once weekly with TCQ (0·3 mg.) in benzene were examined at intervals up to and including the time of tumour formation. In these experiments, groups of four mice were taken and the bound TCQ content of whole skin, rather than epidermal protein, was determined following removal of unbound carcinogen by prolonged extraction with hot solvents.

Control studies on the recovery of TCQ from whole skin by the extraction procedure indicated that because of tissue background absorption, it was necessary for accurate determination to have not less than 1 $\mu$g. of TCQ present in the final benzene extract. This is illustrated in Fig. 2, which shows typical absorption curves (B, C and D) obtained with a final benzene extract of mouse skin to which varying amounts of TCQ had been added. In these experiments, whole skin samples of approximately 5 g. wet weight were used and thus the limit of detection of TCQ was of the order of 0·2 $\mu$g./g. tissue wet weight.

FIG. 2.—Absorption spectra of typical final benzene extracts from control and TCQ-treated mouse skin.

(A) TCQ-treated mouse skin.

(B) Control mouse skin + 1 $\mu$g. TCQ.

(C) Control mouse skin + 2 $\mu$g. TCQ.

(D) Control mouse skin + 4 $\mu$g. TCQ.

Altogether 6 samples of mouse skin were analysed at monthly intervals during continuous weekly treatment with carcinogen. In each case the amount of tissue taken was between 5·6 and 6·8 g. wet weight but no TCQ was detectable in the final benzene extracts. This is illustrated in Fig. 2, which shows a typical absorption curve (A) obtained with the final benzene extract from TCQ treated mouse skin.

*Skin lipid*

Studies on the localisation of TCQ in skin by fluorescence microscopy (Baldwin Chayen and Palmer, 1960) have shown that the compound penetrates the subcutaneous tissue, and that such TCQ can be effectively removed by extraction with lipid solvents. It therefore became necessary to investigate the possibility that TCQ was being extracted in a bound form with lipid.

Mice were painted daily with TCQ in benzene for 3 days, and were killed 24 hours after the last painting. Skins were removed, sliced, frozen in liquid air, and crushed while still in the frozen state. The resultant skin powder was allowed to thaw, homogenised in ethanol (96 per cent) and the total lipid extracted from the skin by the procedure of Hanahan, Dittmer and Warashina, (1957). Phospholipids were separated from the total lipid mixture by precipitation with acetone, and purified by several reprecipitations from light petroleum solution with acetone (Hanahan, Dittmer and Warashina, 1957). Aliquots (16·0 mg.) of the final product were analysed for TCQ and none was found. Control experiments established that the limit of detection of TCQ in phospholipid was approximately 0·1 µg./100 mg. lipid; in this case, the higher sensitivity of the process was due to the lower background absorption.

**Fig. 3.**—Chromatographic fractionation of neutral lipid components of TCQ-treated mouse skin.

Following the separation of phospholipid from the total lipid mixture, the acetone-soluble, neutral lipid was obtained as a yellow oil, which, on examination under ultra-violet light, exhibited strong TCQ-type fluorescence. Portions of this neutral lipid fraction (300 mg.) were fractionated on silicic acid columns by the method of Hirsch and Ahrens (1958) to give cholesterol esters, triglycerides, non-esterified fatty acids, and cholesterol. The separation of lipid from the column was effected by stepwise elution with mixtures of ethyl ether and light petroleum. Fractions were collected and the elution was followed by analysis by weight using the procedure of Craig, Hausmann, Ahrens and Harfenist (1951). More specific identification of each separated lipid component included the titration of non-esterified fatty acids by the method of Dole (1956) and the determination of cholesterol and cholesterol esters by the method of Hanel and Dam (1955).

The separation of the lipid components of neutral lipid from TCQ treated mouse skin is shown in Fig. 3. Detailed analysis of each fraction demonstrated the completeness of separation by this procedure. Examination of the eluted fractions under ultra-violet light showed the presence of TCQ-type fluorescence in tubes 60–75, in which a maximum was found in tube No. 72 (Fig. 3). The TCQ appeared to have separated from all the lipid constituents, although a slight tailing of the non-esterified fatty acid peak into the TCQ peak was observed. This would not appear to be significant, however, since the maxima of the two peaks were well separated. The ultra-violet absorption spectrum of each fraction exhibiting TCQ-type fluorescence was compared with that of authentic TCQ and found to be identical. The identity of the compound was confirmed by means of paper and column chromatography.

*Nucleic acid*

The finding that TCQ is not strongly bound to mouse skin protein or lipid led to experiments designed to investigate the possibility of an interaction with nucleic acid, since evidence for weak binding between hydrocarbons and nucleic acids has been obtained by Booth, Boyland and Orr (1954), and more recently Heidelberger and Davenport (1961) using $^{14}C$ labelled compound, have obtained evidence of strong binding *in vivo* of 1,2 : 5, 6-dibenzanthracene to both deoxyribonucleic acid (DNA) and ribonucleic acid (RNA).

*DNA*.—Mice were painted daily for 3 days with TCQ in benzene and were killed 24 hours after the last painting. The dorsal skin was removed, and subcutaneous tissue separated from the skin by the procedure of Weist and Heidelberger (1953). DNA was isolated from the minced skin by the method of Kirby (1961), and extracted successively with hot ethanol and with hot benzene to remove unbound carcinogen. Analysis of the total DNA sample obtained from the combined skins from 40 mice (70g. wet weight) failed to reveal the presence of any bound TCQ. In control experiments it was shown that limit of detection of TCQ was approximately 1 $\mu$g./total DNA from 35g. wet weight of skin.

*RNA*.—Mice were painted once with TCQ in benzene, killed 24 hours later, and the dorsal skin removed. The skin was separated from subcutaneous tissue by the method of Weist and Heidelberger (1953), and RNA extracted from the minced skin by a modification of the method of Kirby (1956), involving as an extra initial step the addition of a strong phenol solution to a suspension of the chopped skin in water. Overnight, considerable softening of the skin occurred, and the resultant mixture could be successfully homogenised in a Potter-Elvehjem homogeniser. RNA prepared by this method was completely soluble in water and exhibited a pale bluish fluorescence in ultra-violet light. The ultra-violet absorption spectrum in water was found to be identical with that of an RNA sample from the skins of untreated mice, exhibiting a single peak at 259 m$\mu$. TCQ could not be detected in RNA samples (100 mg.) subjected to analysis, and control experiments established a limit of detection of TCQ of approximately 0·1 $\mu$g./100 mg. RNA.

*Subcellular components*

Fiala, Sproul and Fiala (1955) have shown that 3,4-benzopyrene becomes bound to cytoplasmic subcellular components following its application to the skin of day-old mice. The intracellular distribution of TCQ in mouse skin was examined using similar procedures. TCQ in acetone (0·1 mg./ml.) was applied dropwise to cover the entire backs and flanks of day old Strain A mice. At intervals of time varying between 2 and 24 hours after administration of the compound, the animals were killed, and the whole skin stripped off with forceps and homogenised in 0·25 M sucrose. Subcellular fractions were isolated by differential centrifugation, and analysed for TCQ. In a preliminary experiment in which no attempt was made to remove contaminating surface TCQ from the skin, carcinogen was found in all subcellular fractions (Table I). When the experiment

TABLE I.—*Intracellular Distribution of TCQ In Mouse Skin*

| Experiment | Time killed (hours) | TCQ content (μg./g. wt weight original tissue) Nuclei | Mitochondria | Microsomes | Cell sap |
|---|---|---|---|---|---|
| TCQ-treated skin | 5 | 2·9 | 1·4 | 0·3 | 0·3 |
| TCQ-treated skin ; washed with acetone | 2 | — | — | — | — |
| | 5 | — | — | — | — |
| | 24 | — | — | — | — |
| Control skin ; TCQ (10 μg.) added before homogenisation | 5 | 1·4 | 0·9 | 0·6 | 0·3 |

was repeated with acetone washing of the skin before its removal from the body, TCQ could not be detected in any of the subcellular fractions from skin samples taken after intervals of 2, 5 and 24 hours (Table I). This result is in agreement with the finding that TCQ can be removed from the skin of adult mice by solvent extraction. A control experiment was carried out in which TCQ was added to untreated mouse skin immediately preceding homogenisation in order to determine whether association of the carcinogen with subcellular components occurs during the homogenisation of the tissue. From Table I it can be seen that TCQ was found in all subcellular fractions prepared from control skin with added TCQ.

### DISCUSSION

The present study shows that within the limits of detection, TCQ does not bind strongly to epidermal protein at any stage during carcinogenesis. These findings are in contrast to those of Heidelberger (1959) and of Woodhouse (1954) who showed that carcinogenic polycyclic hydrocarbons bind to epidermal protein whilst Daudel *et al.* (1960) have reported similar binding of the carcinogenic angular benzacridines.

The estimation of TCQ in extracts of alkaline hydrolysates of skin was carried out spectrophotometrically utilizing the characteristic peaks of the spectral absorption curve of the carcinogen. Control studies indicated that the limit of detection of TCQ in skin by this procedure was 0·2 μg./g. wet weight of tissue. The levels of the protein-bound metabolites of the carcinogenic polycyclic hydrocarbons and benzacridines were very much greater than this, being of the order of 15 to 40 μg./g. dried epidermal protein.

Recently evidence has been reported suggesting that in certain instances, carcinogens may exert their effect through direct interaction with nucleic acids (Magee and Farber, 1962) whilst Heidelberger and Davenport (1961) have obtained evidence of firm binding *in vivo* of 1,2 : 5,6-dibenzanthracene to both DNA and RNA in skin. Examination of DNA and RNA fractions from TCQ treated skin failed to reveal the presence of any bound TCQ which could not be removed by hot solvent extraction. It must be emphasized however that the radiochemical procedures utilised for assay of 1,2 : 5,6-dibenzanthracene in nucleic acids (Heidelberger and Davenport, 1961) are more sensitive than the assay procedure for TCQ and it is intended to extend the TCQ studies using radioactive carcinogen.

Evidence suggestive of weak tissue binding has been obtained from differential solvent extraction studies on TCQ treated skin (Baldwin, Palmer and Partridge, 1960), and in view of the lipophilic properties of TCQ and the observation that TCQ can form a complex with orthophosphoric acid in a non-aqueous medium (Baldwin, Palmer and Partridge, 1960), the possibility of complex formation with lipid, particularly phospholipid, was investigated. Phospholipid fractions from skin were found to be free from TCQ however, and although the compound was shown to be extracted in the neutral lipid fraction, no evidence was found of binding to any major component of neutral lipid.

Analyses of whole skin for strongly bound TCQ at intervals during carcinogenesis were uniformly negative. Furthermore, investigation of the localization of TCQ in subcellular fractions of mouse skin failed to reveal the presence of strongly bound TCQ, since the distribution of TCQ in cell fractions could be accounted for either by non-specific adsorption or by sedimentation of solid TCQ particles concurrently with cell fractions.

The present findings indicate that within the limits of detection TCQ is not strongly bound to any skin component. In these studies, tissues were examined for bound TCQ utilizing the characteristic light absorption and fluorescent properties of the carcinogen. Therefore the possibility that a metabolite with altered spectral properties is bound to tissue cannot be ruled out. Previous studies on the influence of structural modification on the carcinogenicity of TCQ (Baldwin, Cunningham, Partridge and Vipond, 1962) have indicated, however, that the whole molecule rather than a metabolite is the proximate carcinogen.

The possibility of weak TCQ-tissue interaction still remains to be investigated. The present evidence suggests, however, that any such binding is readily disrupted by the extractive procedures utilized for the isolation of TCQ from tissue.

Studies of the variation of carcinogenicity of TCQ and its derivatives (Baldwin, Cunningham, Partridge and Vipond, 1962) imply the intervention of the 2-, 7-, and 12-positions of the TCQ molecule in TCQ carcinogenesis. A direct, three-point union of TCQ to a cellular constituent at these positions could occur only by covalent bonding. The absence of any indication of strong binding of TCQ to tissue excludes this possibility. As the present study indicates multiple bonding by low-energy bonds is evidently involved.

Generalised intermolecular attraction by van der Waals forces, between the planar carcinogen and a structurally non-specific receptor surface, provides an incomplete interpretation of the evidence. The sensitivity of activity to structural change requires a high structural specificity, not only in the carcinogen, but also in the tissue receptor.

A carcinogen-receptor union by overlap of $\pi$-orbitals of aromatic or heteroaromatic rings in the bonded pair, or by hydrogen bonding of tissue NH or OH to the nitrogen of TCQ, or by interactions of a hexapolar TCQ with ionic or polypolar centres in the tissue are feasible possibilities. The formation of such types of bonds would require a precise stereochemical fit of carcinogen and receptor. Steric hindrance of their formation by clash between functional groups adjacent to the tissue receptor and substituents on the peripheral rings of TCQ, especially at the 2-position (or equivalent 7- and 12-positions), would account for fluctuations in activity in TCQ and its derivatives. Moreover, the general relationship between carcinogenicity and van der Waals radii of single substituents at the 3-position, or multiple substitution at the 3-, 8-, and 13-positions, then becomes explicable.

SUMMARY

1. The epidermal carcinogen, tricycloquinazoline (TCQ) has been examined for possible interactions with skin components.

2. In contrast to other epidermal hydrocarbon carcinogens, no evidence was obtained of firm binding of TCQ with epidermal protein.

3. Examination of whole skin samples at stages during carcinogenesis revealed no binding to any skin component and the lack of strong binding to nucleic acids was confirmed on isolated fractions.

4. In spite of the lipophilic properties of TCQ, no evidence of interaction with phospholipid was obtained and, although the neutral lipid fraction contained TCQ, the carcinogen was shown not to be associated with any particular lipid fraction.

5. The implications of these findings to TCQ carcinogensis are discussed. The present results together with evidence on the variation of TCQ carcinogenicity following structural change suggest that the primary interaction of carcinogen with tissue involves multiple low energy bonding with a highly specific tissue receptor site.

This work was supported by the Nottinghamshire Council of the British Empire Cancer Campaign.

REFERENCES

BALDWIN, R. W., CHAYEN, J. AND PALMER, H. C.—(1960) *Rep. Brit. Emp. Cancer Campgn.*, **38**, 457.

*Idem*, CUNNINGHAM, G. J., PARTRIDGE, M. W. AND VIPOND, H. J.—(1962) *Brit. J. Cancer*, **16**, 276

*Idem*, PALMER, H. C. AND PARTRIDGE, M. W.—(1960) *Rep. Brit. Emp. Cancer Campgn.*, **38**, 454.

BOOTH, J., BOYLAND, E. AND ORR, S. F. D.—(1954) *J. chem. Soc.*, **1**, 598.

CRAIG, L. C., HAUSMANN, W., AHRENS, E. H. Jr. AND HARFENIST, E. J.—(1951) *Analyt. Chem.*, **23**, 1236.

DAUDEL, P., CHENON, B., BUU-HOI, N. P., JACQUIGNON, P., LACASSAGNE, A., PRODI, G., VALLÉE, G., VASQUEZ, R. AND ZAJDELA, F.—(1960) *Bull. Soc. Chim. biol., Paris*, **42**, 135.

DOLE, V. P.—(1956) *J. clin. Invest.*, **35**, 150.

FIALA, S., SPROUL, E. E. AND FIALA, A. E.—(1955) *Proc. Amer. Ass. Cancer Res.*, **2**, 15.

HANAHAN, D. J., DITTMER, J. C. AND WARASHINA, E.—(1957) *J. biol. Chem.*, **228**, 685.

HANEL, H. K. AND DAM, H.—(1955) *Acta chem. scand.*, **9**, 677.

HEIDELBERGER, C.—(1959) " Ciba Foundation Symposium on Carcinogenesis ', London (Churchill) p. 179.

*Idem* AND DAVENPORT, G. R.—(1961) *Acta Un. int. Cancr.*, **17**, 55.

HIRSCH, J. AND AHRENS, E. H. Jr.—(1958) *J. biol. Chem.*, **233**, 311.

KIRBY, K. S.—(1956) *Biochem. J.*, **64**, 405.—(1961) *Biochim. biophys. Acta*, **47**, 18.

MAGEE, P. N. AND FARBER, E., (1962) *Biochem. J.*, **83**, 114.

MILLER, E. C.—(1951) *Cancer Res.*, **11**, 100.

WEIST, W. G. AND HEIDELBERGER, C.—(1953) *Ibid.*, **13**, 246.

WOODHOUSE, D. L.—(1954) *Brit. J. Cancer*, **8**, 346.